# The influence of landscape characteristics on breeding bird dark diversity

**DOI:** 10.1007/s00442-023-05351-8

**Published:** 2023-04-05

**Authors:** Astrid Holm Andersen, Kevin Kuhlmann Clausen, Signe Normand, Thomas Vikstrøm, Jesper Erenskjold Moeslund

**Affiliations:** 1 Department of Biology, Aarhus University, Ny Munkegade 116, 8000 Aarhus C, Denmark; 2 Department of Ecoscience, Aarhus University, C. F. Møllers Allé 8, 8000 Aarhus C, Denmark; 3 BirdLife Denmark, Vesterbrogade 140, 1620 Copenhagen V, Denmark

**Keywords:** Beals’ index, Biodiversity, Conservation, Management, Red list

## Abstract

The exploration of factors and processes affecting biodiversity loss is central to nature management and wildlife conservation, but only recently has knowledge about the absence of species been recognized as a valuable asset to understand the current biodiversity crisis. In this paper, we explore the dark diversity (species that belong to a site-specific species pool but that are not locally present) of breeding birds in Denmark assessed through species co-occurrence patterns. We apply a nation-wide atlas survey of breeding birds (with a 5 × 5 km resolution), to investigate how landscape characteristics may influence avian diversity, and whether threatened and near threatened species are more likely to occur in dark diversity than least concern (LC) species. On average, the dark diversity constituted 41% of all species belonging to the site-specific species pools and threatened and near-threatened species had a higher probability of belonging to the dark diversity than least concern species. Habitat heterogeneity was negatively related to dark diversity and the proportional cover of intensive agriculture positively related, implying that homogeneous landscapes dominated by agricultural interests led to more absent avian species. Finally, we found significant effects of human disturbance and distance to the coast, indicating that more breeding bird species were missing when human disturbance was high and in near-coastal areas. Our study provides the first attempt to investigate dark diversity among birds and highlights how important landscape characteristics may shape breeding bird diversity and reveal areas of considerable species impoverishment.

**Supplementary Information:**

The online version contains supplementary material available at 10.1007/s00442-023-05351-8.

## Introduction

The world’s ecosystems are amidst a global biodiversity crisis (Western 1992; Maxwell et al. 2016; Díaz et al. 2019; IPBES 2019), and recent human-driven changes of natural landscapes have significantly altered previously intact ecological communities (Chase et al. 2020; Plumptre et al. 2021). Consequently, the exploration of factors and processes affecting biodiversity loss is central to contemporary nature management and species conservation (Mazor et al. 2018). While much focus has been on observed diversity, recently knowledge about the absence of species was recognized as an equally valuable source of information as species presences (Pärtel et al. 2011; Lewis et al. 2017). Here, we demonstrate how knowledge of absence in breeding bird species can reveal important knowledge of what determines bird diversity patterns at the 5 × 5 km scale with national extent.

Observed diversity is indeed important in prioritizing conservation efforts but the approach is not always appropriate for comparing biodiversity between ecosystems, regions or taxonomic groups (Pärtel et al. 2013; Moeslund et al. 2017). At any point in time, it is likely that only a part of the species pool (i.e., the ecologically filtered set of species able to inhabit a site) may actually be present in the observed diversity (Pärtel et al. 2013). Thus, the species pool of a given site includes two complementary parts: the observed diversity and the absent (*dark*) diversity (Carmona et al. 2019). While there may be challenges disentangling ‘true’ absences from species that have simply escaped detection (*hidden diversity*) because they are rare or temporarily absent (Pärtel 2014), the *dark diversity* can be defined as the set of species in the region that are capable of dispersing to and establishing in the site, owing to suitable ecological conditions, but which are not currently present (Pärtel et al. 2011). Understanding why these species are absent may reveal novel insights about diversity patterns and drivers of biodiversity loss (Pärtel et al. 2011). For instance, Lewis et al. (2017) reviewed how dark diversity can aid restoration ecology and conservation prioritization by contextualizing the realized diversity (i.e., the species present in a community), to the potential diversity (i.e., those species that might potentially inhabit a site). In this way, the dark diversity can serve as a measure of ‘community completeness’ (Pärtel et al. 2013). Further, Trindade et al. (2020) highlights how dark diversity can support predictions of how biodiversity is likely to respond to global change. Because dark diversity functions as the coupling link between local diversity and the regional species pool, the appearance of species in the dark diversity may suggest either local extinction (species transitioning from the local diversity to the dark diversity) or immigration of new species into the regional species pool (Trindade et al. 2020). Despite its indication of community impoverishment, dark diversity does not necessarily represent an all-negative situation but might also serve as an indication of restoration potential (Pärtel et al. 2011; Moeslund et al. 2017). Evidently, drivers of biodiversity loss may be better understood when focussing not only on observed species richness, but also on species absent from a site where environmental conditions suggest their presence (Pärtel et al. 2011).

Thus far, the majority of studies on dark diversity have focussed on plant communities, where it has been used to identify common characteristics in species that frequently occur in the dark diversity (Riibak et al. 2015; Moeslund et al. 2017), and to investigate the importance of local biotic interactions in determining community richness (Fløjgaard et al. 2020). However, recent studies on biodiversity referring to dark diversity have expanded to include European hoverflies (Miličić et al. 2020), fungi (Valdez et al. 2021), flea assemblages on mammals (Krasnov et al. 2022) and sharks (Boussarie et al. 2018) but to our knowledge no previous attempt has been made to estimate dark diversity in birds.

On a landscape scale, a major driver of bird diversity is landscape composition (the availability and quality of habitats) and landscape configuration (how these habitats are interspersed in space, Dolman 2012; Hinsley and Gillings 2012). The composition of habitats at the landscape scale therefore reflects the availability of vital resources. On the other hand, the landscape configuration provides information about the accessibility of those habitats. For birds, which often use different habitats for different purposes (Fuller 2012b) higher landscape heterogeneity is more likely to support a larger number of species (Fahrig et al. 2011) by improving both landscape *complementation* and *supplementation.* Landscape complementation is defined as the proximity of different habitats containing non-substitutable resources, e.g., nest sites and food sources, and supplementation as the proximity of substitutable resources such as alternative food sources (Dunning et al. 1992). To our knowledge, the effects of these factors on breeding bird dark diversity are largely unknown.

In this paper, we present the first study of breeding bird dark diversity using a nationwide survey of Danish breeding birds to unravel the landscape characteristics driving species absences, and hence, missing avian biodiversity. Denmark houses more than 200 breeding bird species (Dinesen et al. 2016; Vikstrøm and Moshøj 2020), but during the last two centuries the avian fauna has experienced significant declines (Heldbjerg et al. 2020; Meltofte et al. 2021). In Denmark, the newest Red List assessment from 2019 (Flensted and Sterup 2019) shows that 58% of the breeding birds are either regionally extinct (RE), threatened (CR, EN, VU) or near-threatened (NT) (Flensted and Sterup 2019). Thus, less than half of the Danish breeding birds are considered to have stable and viable populations. Further, a decrease in the Red List Index for this species group (Butchart 2008), indicates that the birds are now more threatened than they were at the previous assessment (Flensted and Sterup 2019). We aimed to (1) depict the national pattern of dark diversity among breeding birds in Denmark, and (2) determine which characteristics related to landscape structure and composition are the most important drivers of breeding bird absences in Denmark. In addition, to confirm the role of dark diversity in shaping contemporary avian diversity, we investigated (3) if there were any relationship between the dark diversity of breeding bird species and the degree to which they are nationally threatened (i.e., red list status).

## Methods and material

### Species data

For species data, we used the latest Danish breeding bird atlas survey from 2014 to 2017 (referred to as Atlas III), administered by BirdLife Denmark (Vikstrøm and Moshøj 2020). In this survey, Denmark was divided into 2255 squares of 5 × 5 km forming a regular grid with national coverage (Vikstrøm and Moshøj 2020). Breeding bird distribution data were gathered by voluntary ornithologists over a three-year period, collecting all signs of breeding activity of encountered birds whenever in the field. For each grid cell, a coordinator was appointed to make sure that the grid was thoroughly explored for the occurrence of breeding bird species and that all nature types were visited during the field period (March 1st 2014 to February 28th 2017). To minimize incidents of non-detection, observers were urged to target species that had not yet been detected in a grid cell (Vikstrøm and Moshøj 2020). While this was no guarantee against occasional non-detection of some species, such cases were likely to only have negligible effects on the dark diversity results. The reason for this was that co-occurrence between two species as used in this study was based on all cells in the atlas data, and, that the probability of a species to occur in a given cell, was based on the joint occurrence with all other species observed in the focal community. The probability that an observed species was breeding in a grid cell was registered on a qualitative scale as either possible, probable, or confirmed. This probability was defined from species-specific criteria on breeding behaviour, activity and habitat suitability, and a phenological filter was applied to remove observations of likely migrating birds (Vikstrøm and Moshøj 2020). In the following, only probable and confirmed breeding observations were considered as occurrences and hence referred to as breeding presences. The absence of observations indicative of breeding, including observations labelled as “possible” (i.e., with no clear indications of breeding) were defined as breeding absences. The method used to calculate species pools (see below) assumed an ecological structure in the co-occurrence between species and was sensitive to the frequency of occurrence of individual species and the number of species used to infer the ecological structure (De Cáceres and Legendre 2008). That is, both very rare and very common species can be weakly associated with the inferred ecological structure and thus result in unreliable estimates. To avoid this, we only included grid cells with at least 10 species present and species occurring in ≥ 40 grid cells following De Cáceres and Legendre (2008). Additionally, to reduce the potential effect of species-area relationship on species pool size, coastal grid cells containing > 50% sea cover were excluded from the calculations, as only land was considered potential breeding area. Hereafter, the dataset consisted of 1,721 grid cells and 147 species (the species are listed in Tab. A in Online Resource 1). By considering “possible” breeders absent—as described above—we chose a conservative approach to defining species presences. To make sure that our results were insensitive to this definition, we tested the effect of including the “possible” breeders as presences as well. Naturally, this resulted in an increase in the number of breeding observations (corresponding to 12% in the entire data set), but only had marginal effects on the species pool and dark diversity (both < 5% change). Given the substantial uncertainty related to the actual presences of these possible breeders, we chose to stick with the conservative approach initially taken.

### Site-specific species pools and dark diversity

Dark diversity was originally formulated as the absent portion of the habitat-specific species pool (Pärtel et al. 2011). Thus, to estimate dark diversity, it was necessary to determine which species were ecologically suited for a given habitat (Carmona et al. 2019). For birds, which often exhibit spatial and temporal variation in habitat use (Fuller 2012b), the habitat-specific approach may not be realistic. Here, we therefore refer to site-specific species pools where each site may contain multiple habitats. Further, because birds are highly mobile compared to other taxa for which dark diversity has previously been estimated, we had an additional challenge of determining when a species can actually be considered present. Opposite to immobile species, simply observing a bird at a given site does not necessarily infer its presence as a breeder because individuals frequently traverse unsuitable habitats (Dolman 2012). Thus, the site should at least reflect some degree of association with the species’ breeding requirements and the appropriate landscape configuration, whether it be related to foraging or nesting. For studying birds, the spatial scale (i.e., data resolution) has therefore been coarser than in other studies reflecting bird’s mobility. Here, we considered the grid cell structure of our data set (i.e., 5 × 5 km) suitable to provide a set of “sites” large enough to reflect birds’ mobility and their use of the landscape, but small enough to capture differences in landscape characteristics across larger geographical scales.

Several methods exist to estimate site-specific species pools based on species’ ecological requirements, such as Ellenberg indicators values for plant communities (Ellenberg et al. 1991; Moeslund et al. 2017), species distribution models (de Bello et al. 2016) and co-occurrence patterns (Ronk et al. 2016). Here, we used the principle of co-occurrences to estimate the species pools, which has proven either superior or equally good when compared to other methods in a number of comparative studies (Lewis et al. 2016; de Bello et al. 2016; Ronk et al. 2016). Furthermore, this allowed us to take full advantage of the comprehensive multi-species breeding bird atlas.

Initially, we calculated the probability that a given species would occur in a given grid cell. First, we created a community matrix containing all combinations of grid cells and species presences (coded as 1) or absences (coded as 0). To calculate the aforementioned probabilities, we used Beals’ index (Beals 1984) which is currently the most favoured method for species pool estimations (e.g., Ewald 2002; Moeslund et al. 2017; Munzbergova and Herben 2004). Beals’ index represents the probability of a species occurring at a given site, in this case a given grid cell, based on the overall joint occurrence with other species observed in the focal community (De Cáceres and Legendre 2008). The focal community here refers to the set of species that are observed in a given grid cell. Thus, it can be thought of as a representation of site suitability, assuming an ecological structure in species co-occurrences (Münzbergová and Herben 2004). We used the ‘beals’ function in the ‘vegan’-package (Oksanen et al. 2020) to calculate the Beals’ index value for each species in each grid cell, excluding the focal species from the calculations as recommended by Oksanen et al. (2020). Ideally, occupied grid cells (i.e., where a species was breeding) should represent a higher probability of occurrence, and thus have higher Beals’ index values, than unoccupied grid cells (Münzbergová and Herben 2004). We, therefore, examined this by visually comparing boxplots showing the distribution of Beals’ index values in occupied and unoccupied grid cells, respectively. For all species, the probability of occurrence was higher in occupied grid cells than unoccupied cells (Fig. A in Online Resource 1), which we interpret as a confirmation that the calculated Beals values have suitable explanatory power.

Next, we estimated the regional species pool for each grid cell based on the probabilities described above (i.e., Beals’ index) as follows. The inclusion of a particular species in the species pool was determined by species-specific thresholds (Münzbergová and Herben 2004) represented by the 10^th^ percentile of the Beals’ index values for that species, considering only the index values of occupied grid cells (thresholds presented in Tab. A in Online Resource 1). A range of different thresholds were tested using different percentiles spanning from a very wide inclusion (5^th^ percentile) to a very restrictive one (90^th^ percentile) (Fig. B in Online Resource 1). A slight overrepresentation of rare species obtained from the 10^th^ percentile was favoured over missing actual patterns that were present in the original data, which was the case for the more restrictive thresholds (≥ 15%). Thus, the 10^th^ percentile was considered the best threshold. Furthermore, species that were present in a given grid cell were automatically considered part of the species pool of that site, eliminating the possibility of species being absent from the species pool in grid cells where they were actually observed (Fløjgaard et al. 2020). The Beals values for each species–grid–cell pair were then converted to represent the presence (1) or absence (0) of a species in the species pool based on the species-specific threshold. As a simple assessment of the reliability of species pool predictions, we investigated the linear relationship between the number of observed species in the survey and the number of species in the predicted species pools. This was done using the Pearson correlation coefficient, with the expectation that grid cells with more observed species should give rise to larger species pools which was the case with a high correlation value (Pearson’s *r* = 0.87).

The dark diversity (DD) of each grid cell was calculated by subtracting the number of observed species from the site-specific species pool, thus representing the number of absent species within each grid cell:

$${\text{DD}} = {\text{species}}\;{\text{pool}} - {\text{observed}}\;{\text{species}}$$

To be able to compare different grid cells with different species pools, this was further adjusted to account for differences in species pool size according to the following (Valdez et al. 2021):

$${\text{DD}}_{{\text{adj}}} = \frac{{{\text{DD}}}}{{{\text{total}}\;{\text{no}}{.}\;{\text{of}}\;{\text{species}}\;{\text{in}}\;{\text{species}}\;{\text{pool}}}}$$

This yielded a response variable in which the dark diversity represents the proportion of species in the species pool that are absent in a given grid cell.

Further, to assess the relationship between birds’ dark diversity and their red list status, we calculated the probability for a given species of belonging to the dark diversity (*p*_DD_) as the proportion of times the species was absent (i.e., in the dark diversity) compared to how often it appeared in the species pool (Moeslund et al. 2017):

$$p_{{\text{DD}}} = \frac{{{\text{No}}{.}\;{\text{of}}\;{\text{times}}\;{\text{in}}\;{\text{dark}}\;{\text{diversity}}}}{{{\text{No}}{.}\;{\text{of}}\;{\text{times}}\;{\text{in}}\;{\text{species}}\;{\text{pool}}}}$$

### Explanatory variables

For each grid cell, we calculated four measures reflecting compositional and structural characteristics and one reflecting the degree of human disturbance. We also included distance to the coast as a spatial component. Details about each explanatory variable are explained in the following and summarized in Table 1.

**Table 1:** Details on explanatory variables, their unit and range of values, and accompanying hypotheses with references

Variable	Description	Unit (range)	Hypothesized relationship with dark diversity: …	Reference
Intensive agriculture	Land area covered with permanent or temporary crops	% (0 to 100)	*… positive*	Gil-Tena et al. (2015), Bowler et al. (2019)
Protected nature incl. extensive agriculture	Land area covered by protected nature and extensive agriculture	% (0 to 100)	*… negative*	Cerezo et al. (2011)
Habitat heterogeneity	Number of different habitats	Count (0 to 7)	*… negative*	Dolman (2012)
Forest patchiness	Number of forest patches	Count (0 to 520)	*… negative*	Fischer and Lindenmayer (2002)
Human disturbance	Cumulative line density of transportation networks (roads, paths, railways)	m/km^2^ (373.7 to 42,246.3)	*… positive*	Palomino and Carrascal (2007), McClure et al. (2013), Morelli et al. (2014)
Distance to coast	Euclidean distance to the nearest coastline	m (0 to 47,927)	*… positive*	Ejrnæs et al. (2021)

All data are freely available at the sources presented in the text

#### Intensive agriculture

The agricultural landscape makes up 62% of the land area in Denmark (The Danish Agrifish Agency 2016) and can be considered a highly homogeneous and human modified landscape (Hinsley and Gillings 2012). Here, we use the percentage of land covered by intensive agriculture in each grid cell as one explanatory variable. Data on agriculture were obtained from the national land-use and land-cover (LULC) map, *Basemap03,* which portrays 30 different land cover categories (data from 2018) with a spatial resolution of 10 m (Levin 2019). In this map, temporary crops are distinguished from permanent crops (LULC-codes: *211,000, 212,000* respectively), but for the purpose of this study these were merged to a single variable of intensive agriculture, covering, e.g., rotational grasslands, arable fields and plantations (Levin 2019). Because of the uniformity of the agricultural landscape, it is often associated with low biodiversity (Dudley and Alexander 2017) and thus low suitability for species that are not specialized in the utilization of this particular landscape. The intensification of the agricultural landscape has previously been described as a driver of declines in farmland birds across Europe (Butler et al. 2010; Bowler et al. 2019), reducing the likely presence of specialized species as well (Hinsley and Gillings 2012). Therefore, dark diversity was expected to increase with increasing proportion of land subject to agricultural intensification.

#### Protected nature and extensive agriculture

We used spatially explicit polygon data (last updated in 2018) from Denmark’s environmental portal (Danmarks Miljøportal 2021a, b) to calculate the percentage of land area covered by nature types protected under the Danish nature protection legislation (i.e., Category VI nature: Protected areas with sustainable use of natural resources according to IUCN 2022). These included the open nature types heathland, bogs, grassland, freshwater and coastal meadows as well as most lakes. Because protected nature is a very broad term in Danish national legislation (meaning that it protects against *changes in nature condition*), it may include areas still subject to agricultural interests and management (e.g., fertilization or grazing) that has been part of the previous practice in a given area. Thus, it frequently includes extensive agricultural areas. However, areas of extensive agricultural practices can be valuable habitats for some bird species (Vickery et al. 2001). For that reason, we pooled protected nature and extensive agriculture into a single variable representing sites where the vegetation is more natural than in typical intensive agricultural landscapes. Collectively, these are perceived as nature in its broadest sense (Hinsley and Gillings 2012). Thus, we expected dark diversity to decrease with increasing percentage of land area covered by protected nature or extensive agriculture.

#### Landscape heterogeneity

The compositional heterogeneity of the landscape is a key factor determining the diversity of birds (Dolman 2012). Naturally, the more heterogeneous the landscape the bigger the potential to support a variety of different species (Vickery and Arlettaz 2012). Further, many species have differing requirements for nesting sites and foraging habitats and are thus dependent on a mosaic landscape composition (Dolman 2012). As a measure of compositional heterogeneity, we counted the number of different habitats present in each grid cell. Seven broad habitat types were included: forest and the six nature types protected under the Danish nature protection legislation. It is expected that dark diversity will decrease with increasing heterogeneity.

#### Forest patchiness

As a measure of structural variation in terms of open and closed landscapes, we counted the number of forest patches in each grid cell. Forest patches included all areas categorised as forest in *Basemap03* which were transformed into smoothed polygons and counted. While potentially having a negative effect on certain forest species due to fragmentation (Watson et al. 2002), forest patches can also increase niche diversity, e.g., through edge habitats, which might affect semi-open species and edge-exploiters positively. Thus, forest patchiness is hypothesized to have a net positive effect on overall bird diversity (Fischer and Lindenmayer 2002). Therefore, we expect dark diversity to decrease with increasing number of forest patches.

#### Human disturbance

We calculated the collective density of transportation networks comprising roads, paths and railways as a proxy for human disturbance. These have previously been shown to have negative effects on bird diversity through the creation of barriers, pollution and noise (Palomino and Carrascal 2007; McClure et al. 2013) and are central landscape configuration elements. In addition, road density is a good proxy of human disturbance, which may likewise affect avian diversity locally (Reijnen et al. 1997). Hence, we expected human disturbance to negatively influence the suitability of the landscape for breeding bird species and therefore increase the dark diversity. Data layers on transportation networks were obtained from the Danish public map database *GeoDanmark* (Danmarks Miljøportal 2021a, b), version 2019*,* and the density calculated in meters per square kilometre.

#### Distance to coast

In Denmark, coastal areas are associated with high diversity of red listed species and considered to be less disturbed and modified by humans, thus thought to represent a pristine gradient of human impact on the landscape (Ejrnæs et al. 2021). We, therefore, included distance to the coast as an explanatory variable, by calculating the Euclidean distance from the centroid of each grid cell to the coast (in meters), using the Euclidean distance tool with a 100 m resolution. We expected a decrease in dark diversity with increasing proximity to the coast but expected this effect to be most pronounced within the first few kilometres from the coast (Ejrnæs et al. 2021). Based on this reasoning, we log-transformed this variable.

### Dark diversity of threatened and least concern species

To investigate whether threatened or near-threatened species exhibited a higher probability of occurring in the dark diversity than *least concern* (LC) species, we used the latest national Red List assessment of the breeding population of each species from 2019 (Moeslund et al. 2019). Species in the Red List category *near threatened* (NT) were pooled with the threatened species (i.e., categories *vulnerable* (VU)*, endangered* (EN) and *critically endangered* (CR)) and totalled 65 species, while *least concern* (LC) species totalled 80 species. Two introduced species; *Columba livia* and *Phasianus colchicus* had no category and were therefore excluded from the following analysis.

### Statistical analysis

To test for differences in dark diversity probability between threatened and near threatened species and species of least concern, we used the Wilcoxon Rank Sum Test. To model the relationship between the adjusted dark diversity (DD_adj_) and the landscape characteristics represented by the variables in Table 1, we used a generalized linear model (glm) with binomially distributed error terms and a logit link function. For binomial glms a “weight” is given representing the number of trials (here the species pool size) when the response variable is the proportion of successes (here species in the dark diversity). To test for multicollinearity between explanatory variables, we calculated the Pearson’s correlation coefficient between pairs of variables and later the variance inflation factor (VIF) on the fitted model. The six variables were independent by the ± 0.7 threshold commonly used to indicate correlation (Tab. B in Online Resource 1). The total forest area in each grid cell was initially included as a variable but showed high correlation with other variables and was, therefore, not considered in subsequent analyses. The strongest correlation was found between *intensive agriculture* and *forest patchiness* (pearson = -0.63) followed by *intensive agriculture* and *protected nature including extensive agriculture* (pearson = -0.57). However, using a cut-off value of 5, the VIF results did not indicate multicollinearity between the variables, and thus *forest patchiness* was kept in the final model. Further, we compared models in turn excluding the compositional variables *intensive agriculture* and *protected nature including extensive agriculture,* to a model including both variables (Tab. C in Online Resource 1). Of these, the best model was the one including only *intensive agriculture* (AIC = 11,670, Table 2). However, the difference in AIC between the model excluding *protected nature including extensive agriculture* and the one including both variables was low (ΔAIC = 2, Table 2). Therefore, the variable representing *protected nature and extensive agriculture* was also kept in the final model. To be able to compare the effects of our explanatory variables, these were scaled to a mean of zero and a variance of 1.

**Table 2:** Results of the model selection using Akaike information criterion (AIC). The baseline model includes all variables and is compared to models either excluding (−) or including (+) a given variable

Model	AIC	ΔAIC
All variables	11,672	–
*− Protected nature incl. extensive agriculture*	11,670	2
*− Intensive agriculture*	11,748	76
** + *Y coordinate***	**11,662**	−**10**

Final model given in bold

To test for spatial autocorrelation, we defined neighbourhoods as grid cells within 15 km of a given cell. To do that, we had to remove 3 grid cells from the test as these had no neighbourhood. We then calculated spatial weights and based on that Moran’s *I* of the model residuals using the functions “nb2listw “ and “moran” (both in the spdep package, version 1.2–2). Finally, we ran a permutation test for Moran’s I with 100 permutations using “moran.mc” (same R package as above) to test for statistical significance of the Moran’s *I* value. Based on this test, we detected significant spatial autocorrelation (Moran’s *I* = 0.012, *p* < 0.05). To address this issue, we tested if adding *x* and *y* coordinates (also as second and third order terms) as co-variates to our model had any influence. The *y*-coordinate (linear term) was strongly significant (*p* < 0.001) and hence we kept this in our model. Subsequent re-checking for spatial autocorrelation as described above confirmed that spatial autocorrelation was no longer a concern (Moran’s *I* = 0.008, *p* > 0.05, see also Online Resource 1, Fig. C for residual plot). Hence, the *y*-coordinate was included as a term in the final model, but we do not report or discuss results related to this as this is just a way to account for spatial autocorrelation, and we believe it cannot be interpreted meaningfully. Model performance was assessed through the linear relationship between the Beals estimated dark diversity values and the model-predicted values. Geospatial analyses were performed in ArcMap version 10.8 and statistical analysis in R version 4.0.3.

## Results

**Fig. 1:**
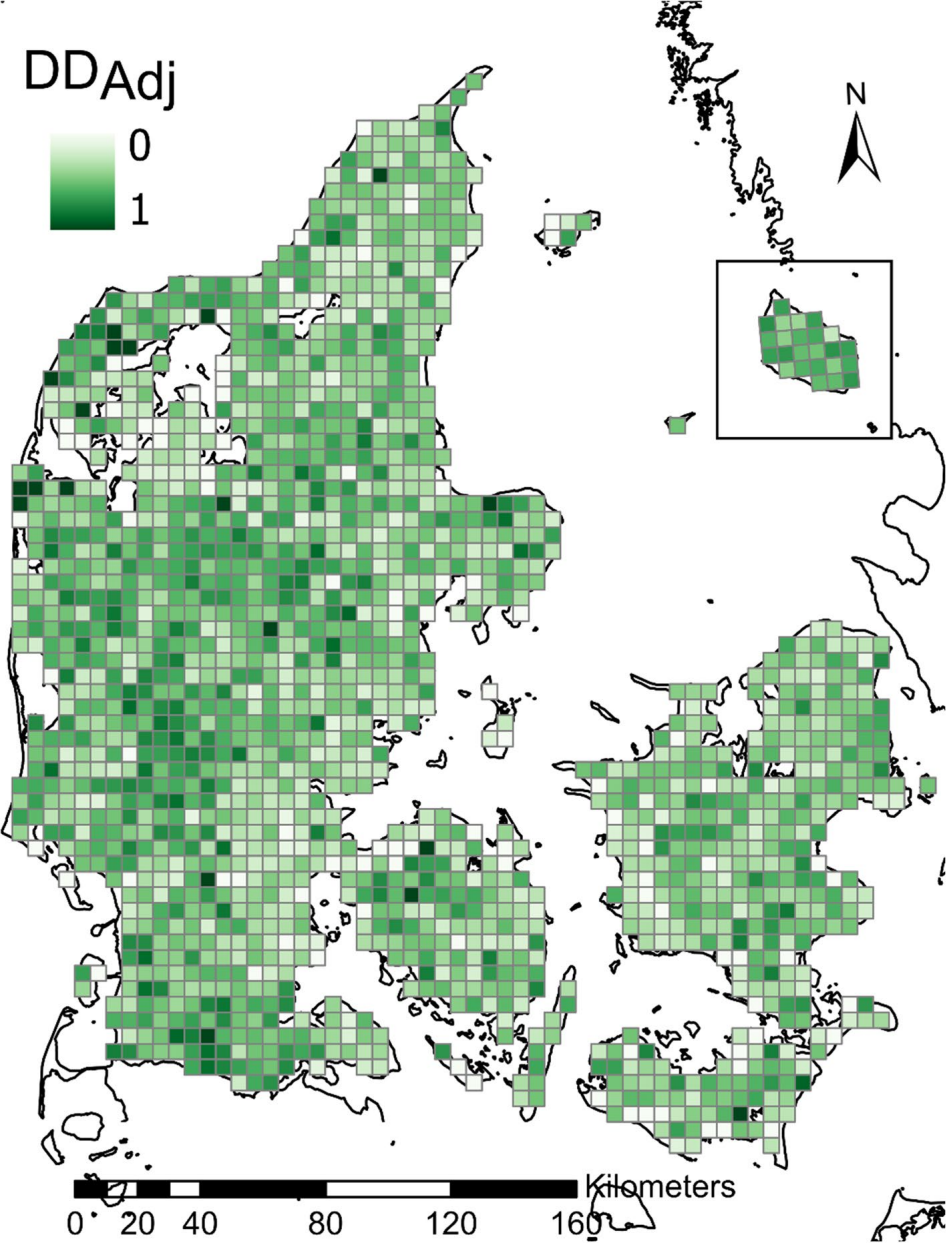
National pattern of the proportion of species in the dark diversity (DD_adj_) for breeding birds in Denmark. Dark diversity is continuous between 0 and 1 with darker greens indicating higher dark diversity

The mean (± SD) size of the species pool of individual cells was 107 species ± 18.69, and the mean number of observed species was 61 ± 15.50. The adjusted dark diversity (DD_adj_) ranged from 0.19 to 0.64 (mean: 0.41 ± 0.06), but low, medium, and high levels of dark diversity were found along the full range of all landscape variables. The national pattern of dark diversity in Danish breeding bird species is shown in Fig. 1.

**Fig. 2:**
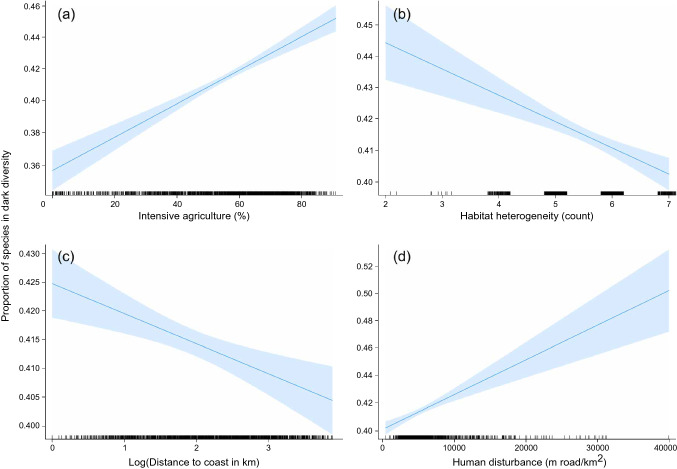
The effect of the four significant explanatory variables on the proportion of species in dark diversity (DD_adj_) predicted by the model: **a** intensive agriculture in percentage of area per grid cell (%), **b** habitat heterogeneity, i.e., the number of different habitats in a grid cell, **c** distance to coast in log(km), and **d** human disturbance, given in meters of road km^−2^. The 95% confidence intervals are represented by the blue shaded area. Note that the y-axis changes between figures

Model results showed that the dark diversity was higher in landscapes with more intensive agriculture and higher human disturbance (Table 3, Fig. 2). In addition, the proportion of species in the dark diversity was lower in landscapes with a larger habitat heterogeneity and when relatively distant to the coast (Table 3, Fig. 2). Mean, range and standard deviation for explanatory variables can be found in Table D in Online Resource 1.

**Table 3:** Results of the generalized linear model with the dark diversity proportion (DD_adj_) as a response and standardized explanatory variables. The 95% confident intervals are also given

Variable	Estimate	Lower CI	Upper CI
*Intensive agriculture*	0.087 (***)	0.069	0.110
*Protected nature incl. extensive agriculture*	0.003 (n.s.)	−0.011	0.018
*Habitat heterogeneity*	−0.026 (***)	−0.036	−0.015
*Forest patchiness*	−0.010 (n.s.)	−0.023	−0.003
*Human disturbance*	0.040 (***)	0.024	0.051
*Distance to coast*	−0.020 (***)	−0.031	−0.009
*Y coordinate*	0.017 (***)	0.008	0.026
Intercept	−0.346 (***)	−0.355	−0.337

^*^p < 0.05, **p < 0.01, ***p < 0.001

Among the six variables investigated, *intensive agriculture* and *human disturbance* had the strongest effects on the proportion of species in the dark diversity, followed by *habitat heterogeneity,* while *distance to coast* had the weakest effect (Table 3)*.* The final model explained 11.7% of the variation in the adjusted dark diversity (with the partial variation explained by the linear y-coordinate term being 0.4%).

**Fig. 3:**
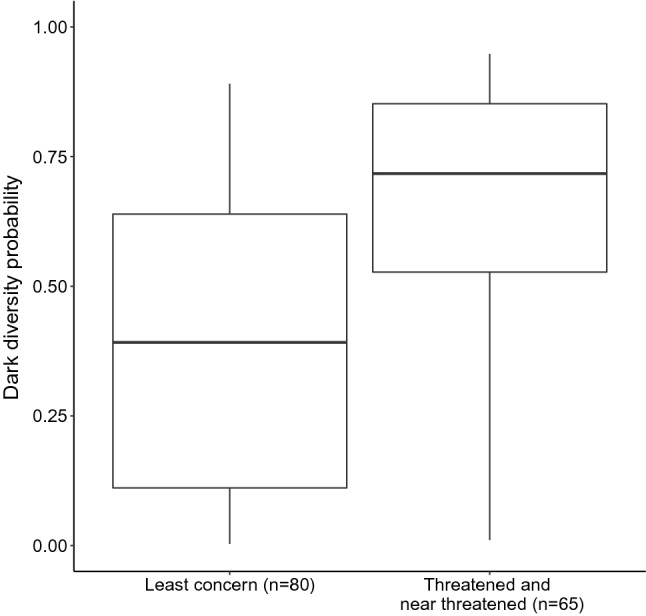
Boxplot of the probability of belonging to the dark diversity for least concern species (*n* = 80) and threatened/near threatened species (*n* = 65)

The median probability that threatened (IUCN red list categories *critically endangered*, CR, *endangered*, EN, and *vulnerable*, VU) and near-threatened (NT) species occurred in the dark diversity was 0.72 (interquartile range, IQR = 0.33). This was significantly higher (*p* ≪ 0.001) than for *least concern* (LC) species; their median probability for belonging to the dark diversity was 0.39 (IQR = 0.53) (Fig. 3).

## Discussion

Because this study estimated the dark diversity relative to the size of the species pool (DD_adj_) it may be interpreted as a measure of species pool completeness. Overall, the dark diversity included at least 19% of the breeding bird species in any given species pool and 30–50% for the majority of grid cells. This indicates that avian communities are rarely fully saturated, which has previously also been suggested for other taxonomic groups (Pärtel et al. 2013; Ronk et al. 2015; Cornell 1999).

### The effects of landscape characteristics

The strongest effect on the proportion of species in dark diversity was the influence of intensively managed agriculture, which, as expected, increased the dark diversity. This is likely associated with substantial agricultural intensification in recent decades, and a resulting decline in diversity and abundance of farmland birds (Schifferli 2000; Donald et al. 2001; Heldbjerg and Fox 2016). Intensive agriculture clearly reduces or deteriorates the suitable breeding and foraging habitats in the cultivated landscapes, and it causes decreased availability of food sources such as insects and other invertebrate prey (Bradbury et al. 2000; Vickery et al. 2001; Benton et al. 2003; Gil-Tena et al. 2015; Bowler et al. 2019; Møller et al. 2021). As a consequence of the more homogenous farmland landscape there is less spatial and temporal variation in available resources, which may lead to increased dark diversity (Herzon and O’Hara 2007; Wuczyński et al. 2011). In addition, many bird species associated with open habitats, such as heathlands and meadows, have likely suffered from large areas of previously uncultivated nature being converted to agricultural land (Dinesen et al. 2016). Likewise, drainage has had major consequences for species associated with wetlands (Thorup 2003; Meltofte et al. 2021). Thus, agricultural intensification has had far-reaching consequences relating to both habitat deterioration of farmland habitats as well as habitat loss for species that are not directly associated with the farmland landscape.

Human disturbance, here in the form of road, path and railway density, related positively to the dark diversity and showed the second strongest relation among the significant effects. The negative impact on diversity was in accordance with the expectation that dark diversity increased in landscapes with higher human disturbance. However, a positive influence of roads has been found for scavenging species benefitting from the road kills of other animals and insects (Lambertucci et al. 2009). Similarly, some bird species may benefit from road verges often having a flower-strip like appearance attracting a diversity of insect prey species (Phillips et al. 2020). But overall, the effect on bird diversity at the scale of this study was expected to be mainly negative. Negative effects of roads on bird populations have been found in several studies (Whited et al. 2000; Forman et al. 2002; Ahmed et al. 2014) and are generally associated with vehicle-caused mortality (Erritzoe et al. 2003), noise pollution (Jaeger et al. 2005), barrier effects, and habitat fragmentation (Forman and Alexander 1998; Ibisch et al. 2016). The presence of humans using roads and paths for recreational purposes can further result in direct avoidance of otherwise suitable habitats (Burger 1995), and high densities of roads often reflect developed areas with limited natural habitats.

Habitat heterogeneity was negatively related to the proportion of species in the dark diversity, indicating that fewer species were missing in more heterogeneous landscapes. This is concordant with other studies showing that landscape heterogeneity promotes species richness (Herzon and O’Hara 2007; Redlich et al. 2018). Two main explanations may account for this. Firstly, different habitats support different species and, thus, cells including multiple habitats can be expected to increase diversity through the accumulation of species with different habitat needs (Fahrig et al. 2011). Secondly, landscape heterogeneity is more likely to support species that have functionally or temporally varying habitat requirements, which is the case, e.g., for species nesting in one habitat but foraging in others (Fuller 2012b; Hinsley and Gillings 2012). This supports landscape configuration as an important driver of breeding bird diversity, by improving both landscape complementation and landscape supplementation in the otherwise homogenous and culturally modified Danish landscape (Dunning et al. 1992). It was, therefore, expected that habitat heterogeneity was a strong determinant of species absences as it is for species presences.

Interestingly, this study did not find a significant relation between dark diversity and the amount of protected nature and extensive agriculture in individual grid cells. This indicates that completeness was not higher in areas with a larger degree of protection than in unprotected areas. However, excluding intensive agriculture as an explanatory variable resulted in a significant negative effect of protected nature and extensive agriculture (Tab. C in Online Resource 1). We believe that this is an indication of the two variables being related to a degree where they represent opposite sides of the same story—namely, the influence of intensive agriculture versus extensively used and protected areas. Alternatively, possible explanations of this pattern might relate to protected areas not providing the intended effects on bird conservation (Albuquerque et al. 2013; Rayner et al. 2014; Brown et al. 2019a, b), or the fact that protected nature only constitutes 10% of the total land area in Denmark (Miljøstyrelsen 2021) and that those areas are often small and fragmented (Meltofte et al. 2021). Consequently, it may be that the effect is negligible for bird diversity at the landscape scale of the current study (Hinsley and Gillings 2012). A further explanation might be that protected nature areas benefit diversity by increasing the species pool, but that proportional dark diversity (the proportion of that species pool being absent) is more or less the same in protected and unprotected areas.

Contrary to our expectations, the number of forest patches in the landscape did not influence the proportion of species in the dark diversity. However, the expected positive effect of many forest patches might have been counterbalanced by a potential negative effect from fragmentation of forest habitats. Previous studies have found that forest fragments, as opposed to large, connected forest areas, have been associated with poorer bird faunas due to edge effects (Andren and Angelstam 1988; Komdeur and Gabrielsen 1993; McIntyre 1995; Fuller 2012a). Such edge effects could potentially influence some species positively by attracting edge-specialists and habitat generalists, despite the smaller forest patches reducing the occurrence of interior species (Fischer and Lindenmayer 2002; Batáry et al. 2014). While previous studies have found that at landscape scales, bird species richness is more affected by the amount of habitat than by fragmentation (Trzcinski et al. 1999; Cerezo et al. 2011; De Camargo et al. 2018), it is possible that the number of forest patches served as important structural elements for some species while leading to fragmentation for others, with no clear directional effect on the overall avian community.

Finally, the relation between dark diversity and distance to the coast contradicted our expectation of a positive relationship, as the adjusted dark diversity was higher near coasts than in interior landscapes. Yet, we failed to identify clear patterns of lower species richness or smaller species pools near the Danish coasts (visual inspection of the relevant GIS maps). It is, therefore, difficult to interpret this result and we urge future studies to explore this further. One potential explanation might be that in many areas the expected “coastal” effect (of more pristine sites) is very local and maybe even limited to a few hundred meters, in which case the spatial resolution of this study (5 × 5 km) was probably too coarse to capture such an effect.

### Threatened and near-threatened species

We showed that the probability for threatened and near-threatened species to belong to the dark diversity was significantly higher than for species categorised as least concern. This indicates that threatened and near-threatened breeding birds are more often missing from otherwise suitable habitats than their least concern counterparts. This again suggests that these species are either slower to establish, disperse more poorly or are more prone to go locally extinct. Given the importance of specific drivers of dark diversity among Danish breeding birds found in this study, these species may be particularly vulnerable to agricultural intensification, homogenisation of landscapes and human disturbance. Future research looking at species-specific variations in dark diversity in relation to drivers of species absences may shed further light on the most important factors effecting the ongoing decline in avian diversity.

### Modelling issues and uncertainties

Uncertainty about observed and dark diversity numbers is often the case for highly mobile species such as birds, for which it can be challenging to determine their presence and whether they are only temporarily absent (cf. *hidden diversity,* see Introduction). However, as the species data in the Atlas III survey were gathered over a three-year period (Vikstrøm and Moshøj 2020) and subject to quality control, we consider it robust to temporary absences, misidentifications and other typical issues for biological datasets. Furthermore, using breeding presences we attempted to ensure that the species were actually living in the given cell and not just visitors, and by considering a relatively large spatial scale (i.e., 5 × 5 km grid cells) we also attempted to account for their high mobility.

Estimates of dark diversity can never be measured exactly as it represents species absences. Currently, only a few well-established methods exist to determine dark diversity (see Introduction), all of them associated with their own set of uncertainties. Thus, determining which one is the better option will depend on the species of interest and the available data. One of the most pronounced uncertainties for methods using co-occurrence principles as in our study is the fact that not all species are associated with certain communities or the ecological structure that is assumed for these methods. Thus, dark diversity estimates should always be interpreted in the light of these uncertainties.

Further, the variables used in our study might be considered rather coarse in terms of the distinction between habitat types, as open habitats were pooled in the single variable of protected nature including extensive agriculture. By extension, the Danish landscape, at the level of available data, was rather monotonous. This was evident from the fact that different levels of dark diversity were found along the full range of all landscape variables, maybe suggesting a limited ability to distinguish between landscape characteristics for different levels of dark diversity. This might partly explain the limited explanatory power of our model and might suggest that future studies should aim for a smaller geographical resolution and a more explicit definition of habitat types.

Additional insight into the determinants of breeding bird diversity might follow from investigating the composition of species in the dark diversity, for example, in relation to functional traits or -groups, taxonomy or different habitats. This could for instance highlight if the observed pattern of dark diversity reflects the changes of species diversity in certain functional guilds such as farmland birds.

### Conclusion

Our study provides the first attempt to quantify the dark diversity of a highly mobile species group and highlights potential important drivers of avian diversity at large geographical scales. We found the most important drivers of breeding bird dark diversity in Denmark to be intensive agriculture and human disturbance, with secondary effects of habitat heterogeneity and distance to the coast. This supports an overall negative impact of human activities on bird diversity, and highlights landscape homogenisation as a major threat to the conservation of bird species. However, the fact that species occur in the dark diversity (and hence in the species pool) is an indication that the negative impact of these factors might not yet be irreversible. For example, bird diversity may benefit from increased heterogeneity, which can be introduced by focussing on the restoration of habitat mosaics in nature planning and management. Given that threatened and near threatened species are more likely to occur in the dark diversity and a large portion of the modern landscape continues to consist of intensively farmed agricultural land, this obviously constitutes an important aspect of future bird conservation in Denmark and probably elsewhere.

## Supplementary Information

Below is the link to the electronic supplementary material.

Supplementary file1 (DOCX 1019 KB)

## Data Availability

The datasets generated during and/or analysed during the current study are available from the corresponding author on reasonable request.

## Code availability

The code generated during the current study is available from the corresponding author on reasonable request.
