# Supplementary material for: The influence of landscape characteristics on breeding bird dark diversity
Source: Oecologia. 2023 Apr 5;201(4):1039–52. doi: 10.1007/s00442-023-05351-8 (PMC10113303; doi:10.1007/s00442-023-05351-8)

**The influence of landscape characteristics on breeding bird dark diversity**

Astrid Holm Andersen, Kevin Kuhlmann Clausen, Signe Normand, Thomas Vikstrøm, Jesper Erenskjold Moeslund^*^

* Corresponding author: [jesper.moeslund@ecos.au.dk](mailto:jesper.moeslund@ecos.au.dk)

Online Resource 1

| **Table A.** Alphabetically ordered list of the 147 breeding bird species in Denmark that have been included in this study. Species names are presented in Latin (lat.) and English (eng.). The list does not include species with less than 40 observations. Additionally, for each species the number of times a species occur in observations and the number of times it occurs in dark diversity (DD) is reported, as well as the species-specific threshold used in the calculation of regional species pools and current Red List category in Denmark (redlist.au.dk, accessed January 2022). |
| --- |

| Family | Species lat. name | Species eng. name | No. Obs. | No. DD | Threshold | Red List category |
| --- | --- | --- | --- | --- | --- | --- |
| Accipitridae | *Accipiter gentilis* | Northern goshawk | 438 | 934 | 0.226 | VU |
|  | *Accipiter nisus* | Eurasian sparrowhawk | 753 | 1095 | 0.377 | VU |
|  | *Buteo buteo* | Common buzzard | 1629 | 364 | 0.776 | LC |
|  | *Circus aeruginosus* | Western marsh harrier | 685 | 919 | 0.335 | LC |
|  | *Haliaeetus albicilla* | White-tailed eagle | 110 | 1201 | 0.057 | NT |
|  | *Milvus milvus* | Red kite | 274 | 1122 | 0.140 | VU |
|  | *Pernis apivorus* | European honey buzzard | 313 | 1004 | 0.168 | NT |
| Acrocephalidae | *Acrocephalus palustris* | Marsh wabler | 1643 | 380 | 0.783 | LC |
|  | *Acrocephalus schoenobaenus* | Sedge wabler | 572 | 1106 | 0.281 | LC |
|  | *Acrocephalus scirpaceus* | Eurasian red wabler | 1345 | 516 | 0.644 | NT |
|  | *Hippolais icterina* | Icterine warbler | 1733 | 375 | 0.811 | VU |
| Aegithalidae | *Aegithalos caudatus* | Long-tailed tit | 807 | 928 | 0.411 | LC |
| Alaudidae | *Alauda arvensis* | Eurasian skylark | 2145 | 23 | 0.984 | NT |
|  | *Lullula arborea* | Woodlark | 356 | 709 | 0.182 | NT |
| Alcedinidae | *Alcedo atthis* | Common kingfisher | 317 | 1134 | 0.169 | VU |
| Anatidae | *Anas crecca* | Common teal | 62 | 1131 | 0.034 | VU |
|  | *Anas platyrhynchos* | Mallard | 1642 | 368 | 0.779 | LC |
|  | *Anas querquedula* | Garganey | 88 | 649 | 0.050 | VU |
|  | *Anser anser* | Greylag goose | 1114 | 665 | 0.536 | LC |
|  | *Aythya ferina* | common pochard | 157 | 706 | 0.089 | VU |
|  | *Aythya fuligula* | Tufted duck | 33 | 927 | 0.177 | NT |
|  | *Bucephala clangula* | Common goldeneye | 49 | 393 | 0.033 | VU |
|  | *Cygnus olor* | Mute swan | 1172 | 757 | 0.561 | LC |
|  | *Mareca strepera* | Gadwall | 184 | 640 | 0.100 | LC |
|  | *Mergus merganser* | Goosander | 81 | 805 | 0.038 | VU |
|  | *Mergus serrator* | Red-breasted merganser | 221 | 755 | 0.104 | VU |
|  | *Somateria mollissima* | Common eider | 161 | 408 | 0.076 | NT |
|  | *Spatula clypeata* | Northern shoveler | 187 | 712 | 0.102 | VU |
|  | *Tadorna tadorna* | Common shelduck | 878 | 1027 | 0.416 | VU |
| Apodidae | *Apus apus* | Common swift | 590 | 1067 | 0.296 | NT |
| Ardeidae | *Ardea cinerea* | Grey heron | 687 | 289 | 0.145 | LC |
|  | *Botaurus stellaris* | Eurasian bittern | 206 | 691 | 0.110 | VU |
| Caprimulgidae | *Caprimulgus europaeus* | European nightjar | 243 | 540 | 0.124 | NT |
| Certhiidae | *Certhia brachydactyla* | Short-toed treecreeper | 515 | 886 | 0.263 | LC |
|  | *Certhia familiaris* | Eurasian treecreeper | 1055 | 709 | 0.523 | LC |
| Charadriidae | *Charadrius dubius* | Little ringed plover | 399 | 1279 | 0.203 | NT |
|  | *Charádrius hiaticula* | Ringed plover | 481 | 854 | 0.206 | VU |
|  | *Vanellus vanellus* | northern lapwing | 1816 | 235 | 0.855 | VU |
| Columbidae | *Columba livia* | Feral pigeon | 279 | 1309 | 0.139 | NA |
|  | *Columba oenas* | Stock dove | 672 | 1017 | 0.341 | LC |
|  | *Columba palumbus* | Common wood pigeon | 2134 | 29 | 0.981 | LC |
|  | *Streptopelia decaocto* | Eurasian collared dove | 1519 | 507 | 0.724 | NT |
| Corvidae | *Corvus corax* | Common raven | 1158 | 735 | 0.561 | LC |
|  | *Corvus cornix* | Hooded crow | 1872 | 226 | 0.879 | LC |
|  | *Corvus corone* | Carrion crow | 211 | 1400 | 0.100 | LC |
|  | *Corvus frugilegus* | Rook | 865 | 891 | 0.413 | LC |
|  | *Corvus monedula* | Western jackdaw | 1396 | 568 | 0.668 | LC |
|  | *Garrulus glandarius* | Eurasian jay | 1132 | 726 | 0.549 | LC |
|  | *Pica pica* | Eurasian magpie | 1823 | 243 | 0.854 | LC |
| Cuculidae | *Cuculus canorus* | Common cuckoo | 1832 | 249 | 0.865 | NT |
| Emberizidae | *Emberiza calandra* | Corn bunting | 1389 | 566 | 0.630 | NT |
|  | *Emberiza citrinella* | Yellowhammer | 2107 | 51 | 0.969 | VU |
|  | *Emberiza schoeniclus* | Common reed bunting | 1678 | 313 | 0.798 | NT |
| Falconidae | *Falco tinnunculus* | Common kestrel | 1123 | 874 | 0.547 | LC |
| Fringillidae | *Acanthis cabaret* | Lesser redpoll | 1018 | 879 | 0.486 | LC |
|  | *Carduelis carduelis* | European goldfinch | 1590 | 445 | 0.757 | LC |
|  | *Spinus spinus* | Eurasian siskin | 197 | 1300 | 0.101 | NT |
|  | *Chloris chloris* | European greenfinch | 2051 | 83 | 0.949 | NT |
|  | *Coccothraustes coccothraustes* | Hawfinch | 731 | 862 | 0.370 | LC |
|  | *Fringilla coelebs* | Common chaffinch | 2135 | 29 | 0.982 | LC |
|  | *Linaria cannabina* | The common linnet | 1796 | 330 | 0.831 | LC |
|  | *Loxia curvirostra* | Common crossbill | 334 | 895 | 0.173 | LC |
|  | *Pyrrhula pyrrhula* | Eurasian bullfinch | 1377 | 541 | 0.668 | LC |
| Gruidae | *Grus grus* | Common crane | 263 | 1068 | 0.131 | LC |
| Haematopodidae | *Haematopus ostralegus* | Eurasian oystercatcher | 908 | 792 | 0.412 | LC |
| Hirundinidae | *Delichon urbicum* | Common house martin | 1847 | 234 | 0.869 | LC |
|  | *Hirundo rustica* | Barn swallow | 2059 | 91 | 0.954 | LC |
|  | *Riparia riparia* | Sand martin | 645 | 1301 | 0.297 | NT |
| Laniidae | *Lanius collurio* | Red-backed shrike | 739 | 1055 | 0.356 | LC |
| Laridae | *Chroicocephalus ridibundus* | Black-headed gull | 262 | 1223 | 0.128 | EN |
|  | *Larus argentatus* | European herring gull | 308 | 966 | 0.141 | LC |
|  | *Larus canus* | Common gull | 410 | 898 | 0.187 | LC |
|  | *Larus fuscus* | Lesser black-backed gull | 81 | 371 | 0.039 | LC |
|  | *Larus marinus* | great black-backed gull | 140 | 238 | 0.075 | LC |
|  | *Sterna hirundo* | Common tern | 129 | 687 | 0.067 | NT |
|  | *Sterna paradisaea* | Arctic tern | 167 | 377 | 0.081 | VU |
|  | *Sternula albifrons* | Little tern | 76 | 325 | 0.038 | VU |
| Locustellidae | *Locustella naevia* | Common grasshopper warbler | 562 | 1100 | 0.288 | LC |
| Motacillidae | *Anthus pratensis* | Meadow pipit | 1241 | 776 | 0.570 | LC |
|  | *Anthus trivialis* | Tree pipit | 1397 | 446 | 0.653 | LC |
|  | *Motacilla alba* | White wagtail | 2082 | 76 | 0.962 | LC |
|  | *Motacilla cinerea* | Grey wagtail | 218 | 1029 | 0.115 | VU |
|  | *Motacilla flava* | Western yellow wagtail | 507 | 1222 | 0.232 | LC |
| Muscicapidae | *Erithacus rubecula* | European robin | 1990 | 108 | 0.927 | LC |
|  | *Ficedula hypoleuca* | European pied flycatcher | 307 | 1458 | 0.160 | VU |
|  | *Luscinia luscinia* | Thrush nightingale | 1054 | 682 | 0.516 | VU |
|  | *Luscinia svecica* | Bluethroat | 226 | 968 | 0.113 | LC |
|  | *Muscicapa striata* | Spotted flycatcher | 1942 | 885 | 0.513 | LC |
|  | *Oenanthe oenanthe* | Northern wheatear | 114 | 1479 | 0.054 | VU |
|  | *Phoenicurus ochruros* | Black redstart | 668 | 1258 | 0.323 | NT |
|  | *Phoenicurus phoenicurus* | Common redstart | 1950 | 153 | 0.912 | LC |
|  | *Saxicola rubetra* | Whinchat | 703 | 992 | 0.343 | LC |
|  | *Saxicola rubicola* | European stonechat | 215 | 825 | 0.102 | LC |
| Panuridae | *Panurus biarmicus* | Bearded reedling | 163 | 692 | 0.090 | LC |
| Paridae | *Cyanistes caeruleus* | Eurasian blue tit | 2055 | 75 | 0.954 | LC |
|  | *Lophophanes cristatus* | European crested tit | 860 | 860 | 0.414 | NT |
|  | *Parus major* | Great tit | 2126 | 28 | 0.979 | LC |
|  | *Periparus ater* | Coal tit | 1603 | 360 | 0.761 | LC |
|  | *Poecile montanus* | Willow tit | 112 | 644 | 0.057 | NT |
|  | *Poecile palustris* | Marsch tit | 1395 | 478 | 0.675 | LC |
| Passeridae | *Passer domesticus* | House sparrow | 1915 | 181 | 0.892 | LC |
|  | *Passer montanus* | Eurasian tree sparrow | 1991 | 109 | 0.926 | LC |
| Phalacrocoracidae | *Phalacrocorax carbo* | Great cormorant | 105 | 853 | 0.053 | LC |
| Phasianidae | *Coturnix coturnix* | Common quail | 702 | 1150 | 0.334 | NT |
|  | *Perdix perdix* | Grey partridge | 842 | 1078 | 0.409 | VU |
|  | *Phasianus colchicus* | Common pheasant | 1778 | 307 | 0.836 | NA |
| Phylloscopidae | *Phylloscopus collybita* | Common chiffchaff | 2146 | 16 | 0.986 | LC |
|  | *Phylloscopus sibilatrix* | Wood wabler | 688 | 993 | 0.346 | LC |
|  | *Phylloscopus trochilus* | Willow wabler | 2031 | 103 | 0.944 | VU |
| Picidae | *Dendrocopos major* | great spotted woodpecker | 1761 | 237 | 0.834 | LC |
|  | *Dryobates minor* | Lesser spottet woodpecker | 52 | 763 | 0.034 | EN |
|  | *Dryocopus martius* | Black woodpecker | 151 | 688 | 0.087 | VU |
|  | *Jynx torquilla* | Eurasian wryneck | 158 | 934 | 0.084 | VU |
|  | *Picus viridis* | European green woodpecker | 508 | 1019 | 0.256 | VU |
| Podicipedidae | *Podiceps cristatus* | Great crested grebe | 489 | 972 | 0.253 | VU |
|  | *Podiceps grisegena* | Red-necked grebe | 656 | 960 | 0.325 | LC |
|  | *Podiceps nigricollis* | Black-necked grebe | 48 | 490 | 0.031 | VU |
|  | *Tachybaptus ruficollis* | Little grebe | 812 | 906 | 0.410 | LC |
| Prunellidae | *Prunella modularis* | Dunnock | 1976 | 149 | 0.922 | LC |
| Rallidae | *Crex crex* | Corn crake | 165 | 1231 | 0.085 | VU |
|  | *Fulica atra* | Eurasian coot | 1352 | 450 | 0.647 | VU |
|  | *Gallinula chloropus* | Common moorhen | 1231 | 645 | 0.599 | VU |
|  | *Porzana porzana* | Spottet crake | 82 | 958 | 0.044 | EN |
|  | *Rallus aquaticus* | Rallus aquaticus | 630 | 988 | 0.321 | LC |
| Recurvirostridae | *Recurvirostra avosetta* | Pied avocet | 210 | 512 | 0.106 | VU |
| Regulidae | *Regulus ignicapilla* | Common firecrest | 168 | 891 | 0.091 | LC |
|  | *Regulus regulus* | Goldcrest | 1451 | 475 | 0.693 | LC |
| Scolopacidae | *Gallinago gallinago* | Common snipe | 525 | 1051 | 0.262 | LC |
|  | *Limosa limosa* | Black-tailed godwit | 44 | 450 | 0.023 | VU |
|  | *Numenius arquata* | Eurasian curlew | 115 | 709 | 0.054 | VU |
|  | *Scolopax rusticola* | Eurasian woodcock | 323 | 834 | 0.172 | LC |
|  | *Tringa totanus* | Common redshank | 554 | 618 | 0.258 | NT |
| Sittidae | *Sitta europaea* | Eurasian nuthatch | 1507 | 393 | 0.722 | LC |
| Strigidae | *Asio otus* | Long-eared owl | 595 | 1266 | 0.296 | LC |
|  | *Bubo bubo* | Eurasian eagle-owl | 105 | 1074 | 0.055 | EN |
|  | *Strix aluco* | Tawny owl | 1023 | 682 | 0.508 | LC |
| Sturnidae | *Sturnus vulgaris* | Common starling | 1980 | 155 | 0.924 | VU |
| Sylviidae | *Sylvia communis* | Common whitethroat | 2173 | 16 | 0.993 | LC |
|  | *Sylvia atricapilla* | Eurasian blackcap | 2108 | 41 | 0.972 | LC |
|  | *Sylvia borin* | Garden warbler | 1904 | 191 | 0.890 | LC |
|  | *Sylvia curruca* | Lesser whitethroat | 2023 | 131 | 0.939 | LC |
| Troglodytidae | *Troglodytes troglodytes* | Eurasian wren | 2136 | 26 | 0.981 | LC |
| Turdidae | *Turdus merula* | Common blackbird | 2159 | 7 | 0.988 | LC |
|  | *Turdus philomelos* | Song thrush | 2045 | 82 | 0.948 | LC |
|  | *Turdus pilaris* | Fieldfare | 214 | 1128 | 0.113 | LC |
|  | *Turdus viscivorus* | Mistle thrush | 1056 | 616 | 0.505 | LC |
| Tytonidae | *Tyto alba* | Barn owl | 172 | 1309 | 0.079 | VU |

| **Table B.** Pearson correlation between pairs of explanatory variables. Critical value considered to be ±0.7. | | | | | | | |
| --- | --- | --- | --- | --- | --- | --- | --- |
|  | Intensive agriculture | Protected nature incl. extensive agriculture | Habitat heterogeneity | Forest patchiness | Distance to coast | Human disturbance | Y coordinate |
| Intensive  agriculture | 1.000 | -0.570 | -0.252 | -0.626 | 0.031 | -0.470 | 0.019 |
| Protected nature incl. extensive agriculture |  | 1.000 | 0.384 | 0.304 | 0.005 | 0.011 | -0.095 |
| Habitat heterogeneity |  |  | 1.000 | 0.152 | -0.216 | 0.048 | -0.083 |
| Forest  patchiness |  |  |  | 1.000 | 0.326 | 0.409 | -0.162 |
| Distance to  coast |  |  |  |  | 1.000 | 0.096 | -0.220 |
| Human  disturbance |  |  |  |  |  | 1.000 | -0.010 |
| Y coordinate |  |  |  |  |  |  | 1.000 |

**Table C.** Results of the generalized linear models excluding intensive agriculture and protected nature incl. extensive agriculture, compared to the final model. The dark diversity proportion (DD_adj_) is the response and explanatory variables are standardized.

| Variable | Final model | Model excluding intensive agriculture | Model excluding  Protected nature incl. extensive agriculture |
| --- | --- | --- | --- |
|  | Estimate | Estimate | Estimate |
| *Intensive agriculture* | 0.087 (***) | (none) | 0.083 (***) |
| *Protected nature incl. extensive agriculture* | 0.003 (n.s.) | -0.045 (***) | (none) |
| *Habitat heterogeneity* | -0.026 (***) | -0.030 (***) | -0.028 (***) |
| *Forest patchiness* | -0.010 (n.s.) | -0.040 (***) | -0.013 (*) |
| *Human disturbance* | 0.040 (***) | -0.001 (n.s.) | 0.037 (***) |
| *Distance to coast* | -0.020(***) | -0.021 (***) | -0.024 (***) |
| *Y coordinate* | 0.017 (***) | (none) | (none) |
| Intercept | -0.346 (***) | -0.347 (***) | -0.346 (***) |
| AIC | 11662 | 11748 | 11670 |
| ^*: p<0.05, **: p<0.01, **: p<0.001^ | | | |

| **Table D.** Details on explanatory variables. Range of values (min, max), mean value and standard deviation is presented for each variable. | | | | | | |
| --- | --- | --- | --- | --- | --- | --- |
|  | Range | |  | Mean |  | SD |
| Intensive agriculture (%) | 0 | 91 |  | 56 |  | 20.02 |
| Protected nature (%) | 0.1 | 90 |  | 13 |  | 9.93 |
| Habitat heterogeneity (count) | 2 | 7 |  | 6 |  | 0.76 |
| Forest patchiness (count) | 0 | 520 |  | 135 |  | 73.67 |
| Human disturbance (m/km^2^) | 906 | 39,852 |  | 5,297 |  | 3,871.34 |
| Distance to coast (km) | 0 | 48 |  | 10 |  | 9.66 |

**Figure A.** Boxplots of Beals values in occupied (light grey) and unoccupied (dark grey) grid cells for all 147 bird species. Note that the range of values on the y-axis changes. Continued on the next page.


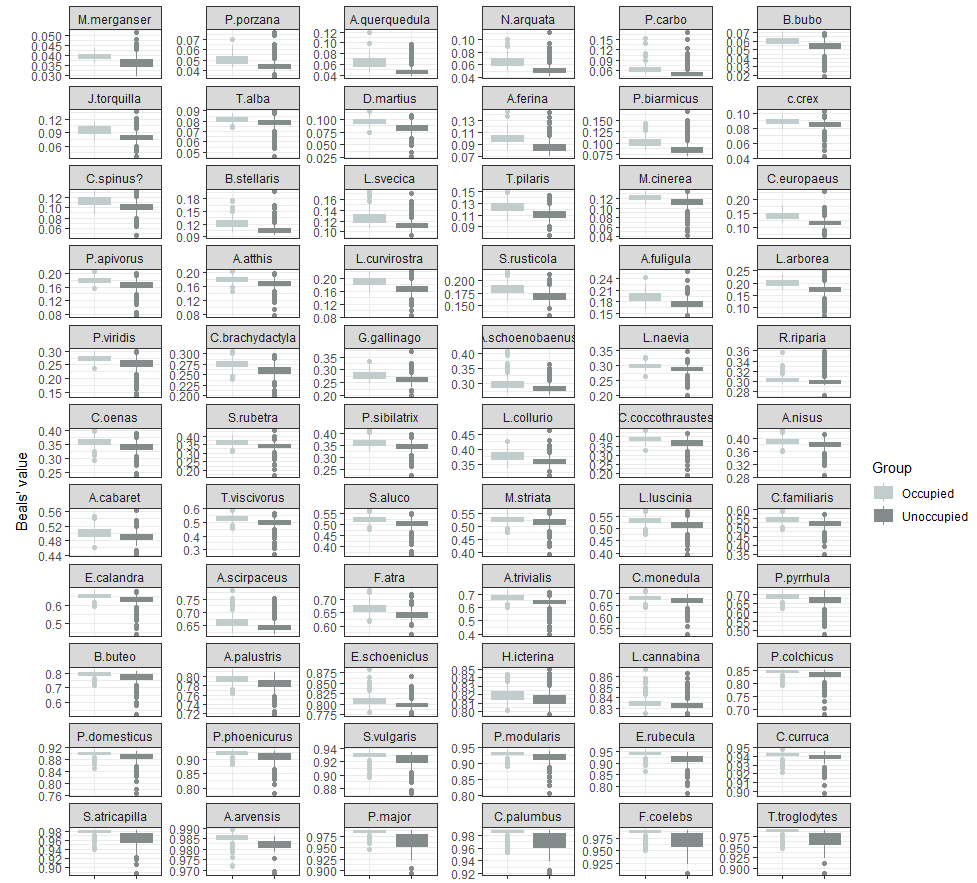


**Figure A (continued).** Boxplots of Beals values in occupied (light grey) and unoccupied (dark grey) grid cells for all 147 bird species. Note that the range of values on the y-axis changes.


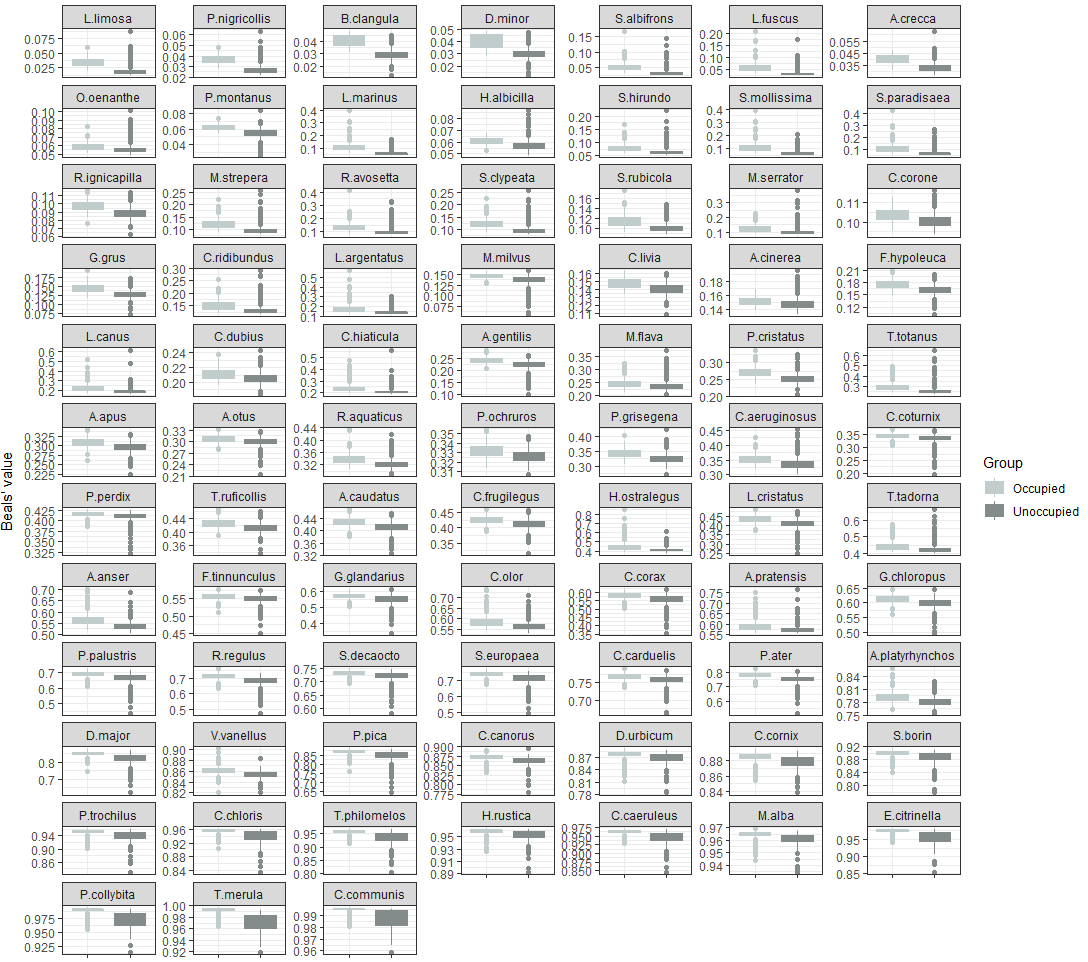


**Figure B.** Change in the number of times a species occurs in predicted species pools at different Beals thresholds versus the number of times they are observed in the survey. Each point represents a species.


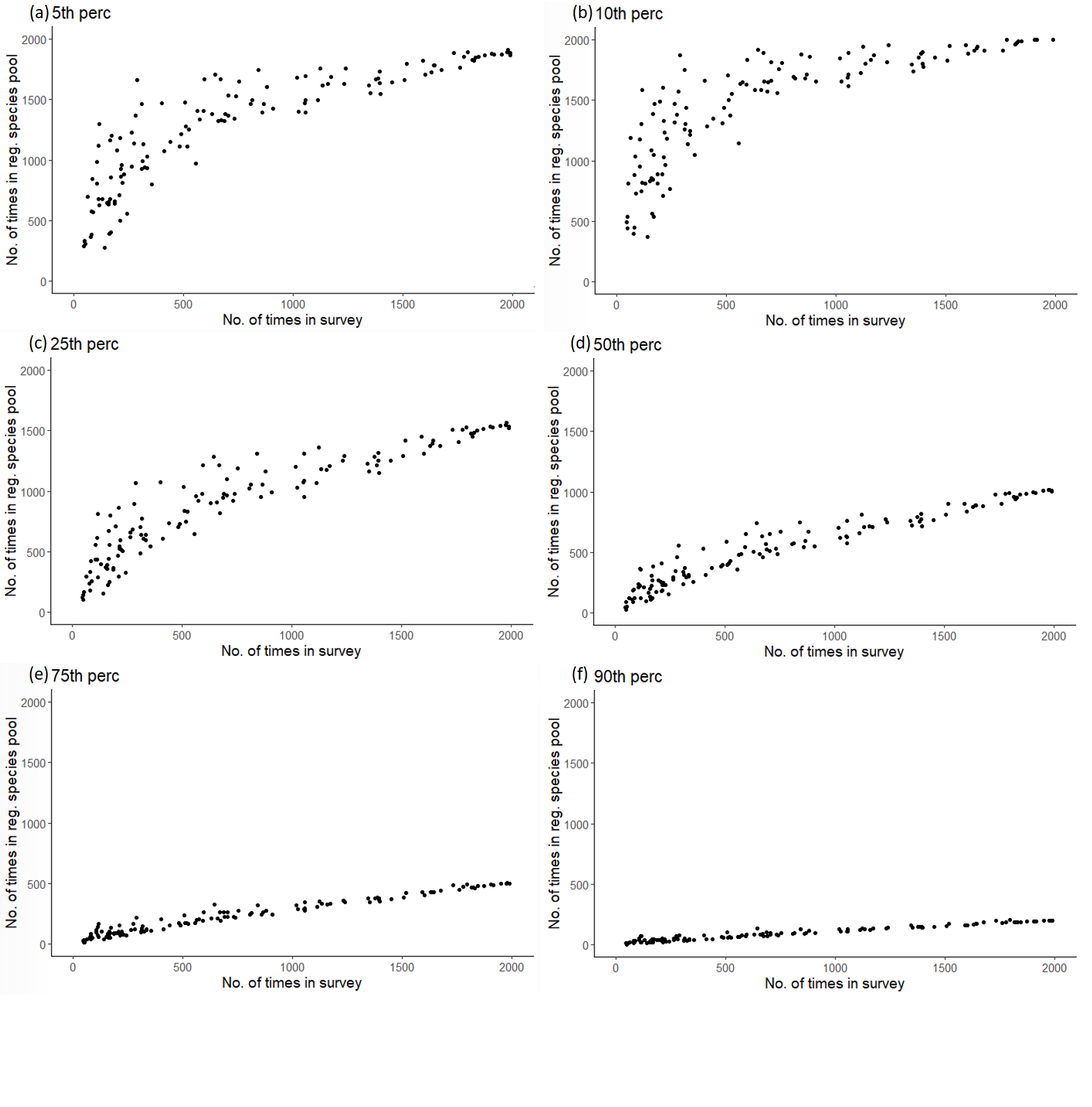


**Figure C.** Residual plot of spatial autocorrelation analysis.


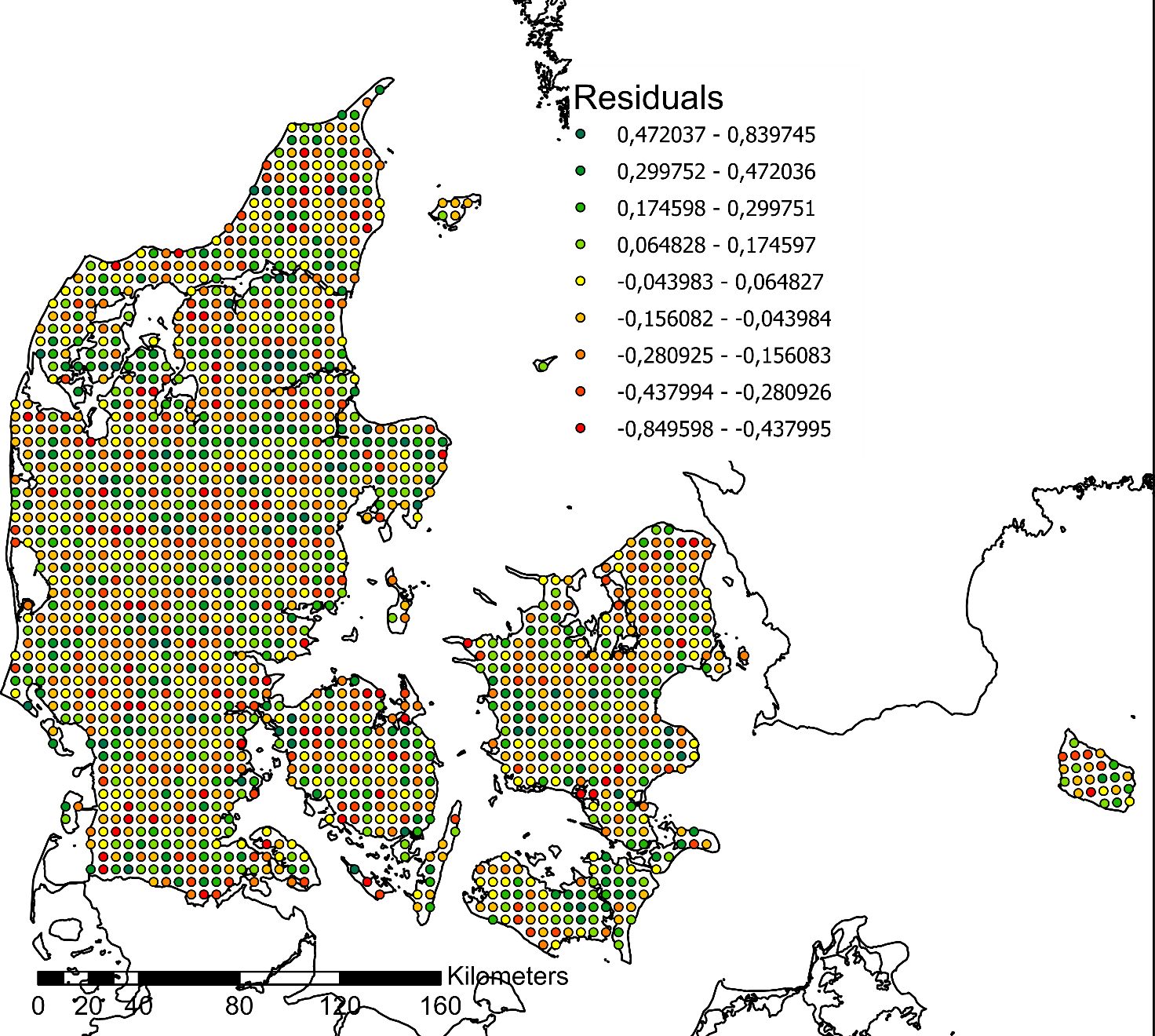

Supplement: Supplementary file 1 — Supplementary file1 (DOCX 1019 KB) [file 442_2023_5351_MOESM1_ESM.docx]
